# Effects of Low Doses of Bisphenol A on the Metabolome of Perinatally Exposed CD-1 Mice

**DOI:** 10.1289/ehp.1205588

**Published:** 2013-02-21

**Authors:** Nicolas J. Cabaton, Cécile Canlet, Perinaaz R. Wadia, Marie Tremblay-Franco, Roselyne Gautier, Jérôme Molina, Carlos Sonnenschein, Jean-Pierre Cravedi, Beverly S. Rubin, Ana M. Soto, Daniel Zalko

**Affiliations:** 1 Institut National de la Recherche Agronomique (INRA), UMR1331, TOXALIM (Research Centre in Food Toxicology), Toulouse, France; 2 Université de Toulouse, UMR1331, Toulouse, France; 3 Department of Anatomy and Cellular Biology, Tufts University School of Medicine, Boston, Massachusetts, USA

**Keywords:** bisphenol A, endocrine disruptor, fetal origins of adult disease, low dose, metabolomics, metabonomics, NMR fingerprints, partial least-squares discriminant analysis (PLS-DA), perinatal exposure, toxicology

## Abstract

Background: Bisphenol A (BPA) is a well-known endocrine disruptor used to manufacture polycarbonate plastics and epoxy resins. Exposure of pregnant rodents to low doses of BPA results in pleiotropic effects in their offspring.

Objective: We used metabolomics—a method for determining metabolic changes in response to nutritional, pharmacological, or toxic stimuli—to examine metabolic shifts induced *in vivo* by perinatal exposure to low doses of BPA in CD-1 mice.

Methods: Male offspring born to pregnant CD-1 mice that were exposed to vehicle or to 0.025, 0.25, or 25 µg BPA/kg body weight/day, from gestation day 8 through day 16 of lactation, were examined on postnatal day (PND) 2 or PND21. Aqueous extracts of newborns (PND2, whole animal) and of livers, brains, and serum samples from PND21 pups were submitted to ^1^H nuclear magnetic resonance spectroscopy. Data were analyzed using partial least squares discriminant analysis.

Results: Examination of endogenous metabolic fingerprints revealed remarkable discrimination in whole extracts of the four PND2 newborn treatment groups, strongly suggesting changes in the global metabolism. Furthermore, statistical analyses of liver, serum, and brain samples collected on PND21 successfully discriminated among treatment groups. Variations in glucose, pyruvate, some amino acids, and neurotransmitters (γ-aminobutyric acid and glutamate) were identified.

Conclusions: Low doses of BPA disrupt global metabolism, including energy metabolism and brain function, in perinatally exposed CD-1 mouse pups. Metabolomics can be used to highlight the effects of low doses of endocrine disruptors by linking perinatal exposure to changes in global metabolism.

Bisphenol A (BPA) is manufactured at a rate of > 3.1 million tons/year; thus, exposure to this compound is ubiquitous. Its possible impact on human health is reflected in recent worldwide regulatory legislation. For example, the Canadian authorities, followed by the European Union, have recently banned the use of BPA in infant feeding bottles, a landmark move to safeguard the health of infants and the general population (Asimakopoulos et al. 2012; European Commission 2011). Nevertheless, BPA is still widely used in polycarbonates, epoxy resins, paints, lacquers, and medical devices. BPA is also used as a base compound for the manufacture of flame retardants, brake fluids, and thermal papers (Fernandez et al. 2007). BPA monomers migrate out of these products and contaminate, for example, food, beverages, and intravenous infusions, (Goodson et al. 2004). Although the main route of contamination is through ingestion, the transdermal route could also contribute to BPA exposure in humans when direct contact with BPA (free monomer) occurs (Zalko et al. 2011). Detectable levels of BPA were present in urine samples of > 92% of Americans tested in the 2003–2004 National Health and Nutrition Examination Survey (Calafat et al. 2008). Higher exposure levels were found in children and adolescents compared with adults. Of particular concern is the finding of high levels of BPA in premature infants being treated in neonatal intensive care units (Calafat et al. 2009). BPA has also been detected in maternal and fetal plasma, in human placenta, and in the milk of nursing mothers (Calafat et al. 2006; Sun et al. 2004). Animal studies have highlighted the estrogenic effects of BPA (vom Saal et al. 2007), although BPA is considered by some to be a weak estrogen due to its low potency compared with estradiol in reporter gene assays involving nuclear receptors (Blair et al. 2000). However, recent work has shown that BPA can be as potent as or more potent than estradiol in promoting some estrogenic activities (Alonso-Magdalena et al. 2006, 2012). BPA can also bind to membrane receptors (G protein-coupled receptor 30 and the membrane form of estrogen receptor-α) to produce effects similar to those of estradiol (Thomas and Dong 2006; Welshons et al. 2006; Wozniak et al. 2005).

Perinatal BPA exposure has been reported to decrease fertility and fecundity in female CD-1 mice (Cabaton et al. 2011) and to decrease fertility in male offspring of exposed rats (Salian et al. 2011). Additional effects of perinatal BPA exposure include masculinization of behaviors and brain structures in female CD-1 mice (Richter et al. 2007; Rubin et al. 2006). Exposure to BPA through placenta and milk has been shown to increase early adipose storage and adipogenesis in a sex-specific and dose-dependent manner in rats and mice, with consequences on body weight later in life (Rubin and Soto 2009; Somm et al. 2009). BPA exposure has been linked to altered glucose homeostasis in pregnant female rodents and their male offspring (Alonso-Magdalena et al. 2010) and has been postulated to be a contributing factor in predisposing populations to the development of obesity and diabetes later in life (Heindel and vom Saal 2009; vom Saal et al. 2012).

The aim of metabonomics is to measure the “global, dynamic metabolic response of living systems to biological stimuli” (Nicholson and Lindon 2008). Metabolomics have furthered our understanding of drug toxicology while complementing more traditional approaches (Coen 2010). The integration of metabolomics and conventional toxicological studies is expected to provide valuable information for risk assessment of endocrine disruptors (EDs) such as BPA. Metabolic fingerprints based on nuclear magnetic resonance (NMR) spectroscopy, combined with appropriate statistical methods, could detect slight changes in the metabolome of cells, tissues, or organisms exposed to EDs, opening the way to examine whether exposure to an ED results in global alterations of metabolism and whether these changes persist after cessation of exposure.

Metabolomic data are characterized by a large number of variables and by high correlations among these variables. Multivariate methods, such as principal components analysis (PCA) and partial least squares discriminant analysis (PLS-DA), which aim to solve both problems mentioned above, are thus the methods of choice for analyzing metabolomic data. PCA is used to determine the variation in the data set, regardless of the origin of this variation. PLS-DA is an alternative to PCA that allows discriminating observations according to classes defined *a priori*. Besides, PLS-DA is preferred to PCA for sample discrimination because the dimension reduction provided by PLS is explicitly guided by variability among groups, whereas PCA can identify only gross variability directions and is incapable of distinguishing variability that occurs among groups and within groups (Quintás et al. 2012). When more than two groups are included in the analysis, PLS-DA is more appropriate than PCA (Lindon et al. 2004).

We hypothesized that the global metabolism of mice perinatally exposed to BPA may be disrupted. The present study was designed to test this hypothesis in the outbred CD-1 mouse, a common animal model in BPA studies. Rodent strains vary in their susceptibility to low-dose BPA exposure (Richter et al. 2007). Rodent placentation at late stages of gestation is histologically close to human placentation (Zalko et al. 2003), and the mouse and rat have been shown to be excellent models for understanding the human syndrome observed in the offspring of mothers exposed to diethylstilbestrol during pregnancy (Vandenberg et al. 2009). Previous work using CD-1 mice has shown that, following transplacental transfer, fetuses become exposed to their mother’s BPA burden and BPA metabolites (Markey et al. 2001; Wadia et al. 2007; Zalko et al. 2003). The body of literature clearly demonstrates multiple targets affected by low doses of BPA, including metabolism, especially when the exposure occurs at critical periods of development (Vandenberg et al. 2012).

In the present study, CD-1 fetuses and neonates were exposed to low doses of BPA [0.025, 0.25, or 25 µg BPA/kg body weight (bw)/day] administered to their mothers from the end of gestation day (GD) 8 through day 16 of lactation. Female offspring were examined for reproductive outcomes and found to have decreased fertility and fecundity (Cabaton et al. 2011), whereas the male offspring were examined for changes in global metabolism by assessing ^1^H NMR profiling and analyzing the data using multivariate statistics. Male offspring were examined on postnatal day (PND) 2 (whole body) and on PND21. For the latter group, samples included the liver (the main metabolizing organ), serum (circulating metabolites), and the brain, given the fact that recent studies have suggested that perinatal exposure to low doses of BPA could have persistent effects on brain structure, function, and behavior in rodents (Richter et al. 2007).

## Materials and Methods

*Chemicals*. Dimethylsulfoxide [DMSO; Chemical Abstracts Service Registry Number (CASRN) 67-68-5)], bisphenol A (4,4´-dihydroxydiphenyldimethylmethane; CAS 80-05-7; product #239658, lot #03105ES; purity ≥ 99%), and butylated hydroxytoluene (BHT) were purchased from Sigma Chemical Company (St. Louis, MO, USA). Stock purity of BPA was confirmed as described previously (Cabaton et al. 2011). We purchased acetonitrile from Scharlab SL (Sentmenat, Spain), and deuterium oxide (D_2_O) and sodium 3-trimethylsilyl-2,2,3,3-tetradeuteriopropionate (TMSP) from Euriso-top (Saint-Aubin, France).

*Animals*. Twelve-week-old female CD-1 mice and proven breeder male mice (Charles River Laboratories, Wilmington, MA, USA) were maintained in temperature- and light-controlled (14 hr/10 hr light/dark cycle) conditions at the Human Nutrition and Research Center animal facility (Tufts University), a facility approved by the Association for Assessment and Accreditation of Laboratory Animal Care. All experimental procedures were approved by the Tufts University–Tufts Medical Center Institutional Animal Care and Use Committee. The animals were treated humanely and with regard for alleviation of suffering. Water (glass bottles) and food (Harlan Teklad 2018; Harlan Laboratories Inc., Indianapolis, IN, USA) were supplied *ad libitum.* The estrogenicity of food lots was measured by the E-SCREEN assay (Soto et al. 1992) and was also tested independently by W.V. Welshons (University of Missouri-Columbia, Columbia, MO, USA) (Welshons et al. 1990), confirming a negligible estrogenic activity (< 20 pmol of estrogen equivalents per gram). Cages, water, and bedding all tested negligible for estrogenicity by the E-SCREEN assay (Soto et al. 1992).

Mice were allowed to acclimatize for 5 days before being mated. The morning on which a vaginal plug was detected was considered GD1. On the evening of GD8, dams were implanted subcutaneously with Alzet osmotic pumps (model 2004; Alza Corp., Palo Alto, CA, USA) following the manufacturer’s recommendations. Pumps were designed to deliver vehicle alone (50% DMSO in water) or one of three doses of BPA—0.025, 0.25, or 25 µg/kg bw/day—based on the mother’s body weight at GD6. These pumps continued to release at a constant rate (0.25 µL/hr) until day 16 of lactation (PND16); however, because the BPA dose was calculated based on the weight of the mother at GD6 and because maternal body weight increased from this point throughout pregnancy, the actual delivered dose of BPA decreased as pregnancy progressed. More than 90% of the dams delivered naturally; the F1 litters were culled to eight pups each, with no differences in sex ratio between treatment groups on the day after birth. Litters were weaned on PND21.

*Experimental design.* On PND2, one F1 male mouse was randomly chosen from each litter and euthanized by decapitation (control, *n* = 20; 0.025 µg BPA, *n* = 18; 0.25 µg BPA, *n* = 14; 25 µg BPA, *n* = 11). On PND21, one F1 male mouse was randomly chosen from each litter and euthanized by CO_2_ asphyiation. Blood samples (control, *n* = 11; BPA 0.025 µg/kg, *n* = 12; 0.25 µg BPA, *n* = 14; BPA 25 µg/kg, *n* = 12) and brain and liver samples (control, *n* = 11; BPA 0.025 µg/kg, *n* = 11; 0.25 µg BPA, *n* = 13; BPA 25 µg/kg, *n* = 14) were collected from each animal.

*Sample preparation for ^1^H NMR spectroscopy.* Trunk blood was collected into serum tubes (Sarstedt, Newton, NC, USA). Blood samples were centrifuged for 5 min at 10,000 × *g* and 20°C. Serum was collected into microtubes and stored at –20°C. For analysis, serum samples (100 µL) were diluted with 600 µL D_2_O and centrifuged at 5,000 × *g* for 10 min before they were placed in 5-mm NMR tubes.

For liver, brain, and whole-pup samples, extraction procedures were derived from Folch et al. (1951) and from the method described by Waters et al. (2002). Samples of tissue (100 mg liver, whole brain, and whole pup) were homogenized using a Polytron PT2100 homogenizer (Kinematica, Lucerne, Switzerland) in acetonitrile/H_2_O (50/50, vol/vol) containing 0.1% BHT in an ice-water bath. Homogenates were centrifuged at 5,000 × *g* for 10 min at 4°C, and the supernatants were removed and lyophilized. The lyophilisates were reconstituted in 600 µL D_2_O containing 0.25 mM TMSP (as a chemical shift reference at 0 ppm). The reconstituted solutions were transferred to NMR tubes.

*^1^H NMR analyses*. All ^1^H NMR spectra were obtained on a Bruker DRX-600-Avance NMR spectrometer (Bruker, Wissembourg, France) operating at 600.13 MHz for ^1^H resonance frequency using an inverse detection 5 mm ^1^H-^13^C-^15^N cryoprobe attached to a cryoplatform (the preamplifier cooling unit).

The ^1^H NMR spectra were acquired at 300K using the Carr-Purcell-Meiboom-Gill (CPMG) spin-echo pulse sequence with pre-saturation, with a total spin-echo delay (2nτ) of 100 msec to attenuate broad signals from proteins and lipoproteins. A total of 128 transients were collected into 32,000 data points using a spectral width of 12 ppm, a relaxation delay of 2.5 sec, and an acquisition time of 2.28 sec. Prior to Fourier transformation, we applied an exponential line-broadening function of 0.3 Hz to the free induction decay.

To confirm the chemical structure of metabolites of interest, we performed two dimensional (2D) ^1^H-^1^H COSY (correlation spectroscopy) and 2D ^1^H-^13^C-HSQC (heteronuclear single quantum coherence spectroscopy) NMR experiments on selected samples.

*Data reduction and multivariate statistical analyses*. All NMR spectra were phased and baseline corrected, then data was reduced using AMIX (version 3.8; Bruker Analytik, Rheinstetten, Germany) to integrate 0.01 ppm–wide regions corresponding to the δ 10.0–0.5 ppm region. The δ 5.1–4.5 ppm region, which includes water resonance, was excluded. A total of 791–861 NMR buckets were included in the data matrices. To account for differences in sample amount, each integrated region was normalized to the total spectral area. Multivariate analyses were used to study the effect of the treatment (DMSO or 0.025 µg, 0.25 µg, or 25 µg BPA/kg bw/day) on the metabolome. We first performed PCA to reveal intrinsic treatment-related clusters and detect eventual outliers. PLS-DA was then used to model the relationship between group and spectral data. PLS-DA is similar to PCA but uses discriminant variables that correlate to class membership. Before analysis, we used orthogonal signal correction filtering (Wold et al. 1998) to remove variation not linked to the treatment (physiological, experimental, or instrumental variation). Filtered data were mean centered and scaled (unit variance or Pareto scaling). For the figures, we used Hotelling’s T^2^ statistics to construct 95% confidence ellipses. The R^2^Y parameter represents the explained variance. Seven-fold cross-validation was used to determine the number of latent variables to include in the PLS-DA model and to estimate the predictive ability (Q^2^ parameter) of the adjusted model. In addition, the robustness and validity of the PLS-DA results were calculated using a permutation test (200 permutations). Discriminant variables were determined using VIP (variable importance in the projection), an appropriate quantitative statistical parameter ranking the descriptors according to their ability to discriminate different doses. We used this global measure of the influence of each variable on the PLS components to derive a subset of the most important metabolites for the separation of experimental groups. We then used the Kruskal–Wallis test to determine which metabolites were significantly different between the groups. SIMCA-P software (V12; Umetrics AB, Umea, Sweden) was used to perform the multivariate analyses.

## Results

We observed no statistical differences in the weight of PND2 pups (whole body) or PND21 livers or brains. PCA results for the NMR spectral data sets [aqueous extracts of PND2 pups (whole body) and serum, liver, and brain from PND21 male mice] are available in Supplemental Material, Figures S1–S4 (http://dx.doi.org/10.1289/ehp.1205588). The PCA score plots allowed a primary separation between groups. We then used a supervised PLS-DA model to further investigate the differences between groups.

**Figure 1:**
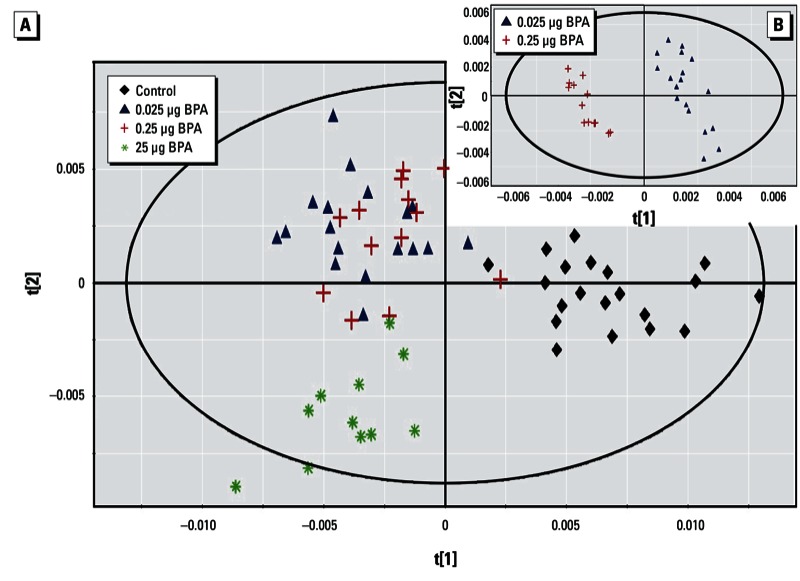
Two-dimensional PLS-DA score plot of integrated ^1^H NMR spectra of PND2 whole body extracts. (*A*) PLS‑DA results for all treatment treatment groups (control, *n* = 20; 0.025 µg BPA, *n* = 18; 0.25 µg BPA, *n* = 14; and 25 µg BPA, *n* = 11) (first and second latent variable of three components: R^2^Y = 71.5% and Q^2^ = 0.557). (*B*) PLS‑DA results for 0.025 µg BPA and 0.25 µg BPA taken separately (first and second latent variable of three components: R^2^Y = 73.1% and Q^2^ = 0.731).

*PND2 pups (whole body).* For the four experimental groups, the analysis generated a PLS-DA model with three latent components, characterized by a faithful representation of the data (R^2^Y = 71.5%) and, more important, by a good cumulative predictive capacity (Q^2^ = 0.557) (Figure 1A). The score plot of the PLS-DA showed a clear separation between the control group and the BPA groups; 83 variables had a VIP value > 1.0 (arbitrary threshold), and the median of 71 buckets was statistically different by the Kruskal–Wallis test. These differences corresponded to 20 metabolites, according to 2D NMR spectra identification. Endogenous metabolite variations induced by BPA exposure (25 µg) in PND2 pups (whole body samples) showed increases in lactate, glucose, cholines, creatine, and glycine compared with control samples. Conversely, we observed decreases in valine, leucine, isoleucine, and lysine (Table 1). In addition, taken separately and using a pairwise comparison, all groups could be successfully discriminated, including the 0.025 µg BPA and 0.25 µg BPA groups (Table 2; Figure 1B). The latter analysis generated a PLS-DA model with three latent components, characterized by a very faithful representation of the data (R^2^Y = 98.0%), with > 90% variability explained along axis 1, and by a very good cumulative predictive capacity (Q^2^ = 0.731). This analysis identified 13 metabolites that contributed to the difference in metabolic profiles between 0.025 µg BPA and 0.25 µg BPA groups by VIP (> 1) and Kruskal–Wallis test (Table 1).

**Table 1:** Endogenous metabolite variations induced by BPA exposure (25 µg) in PND2 pups and in serum, liver, and brain of PND21 mice compared with controls.

Metabolites	1H NMR chemical shifts δ (ppm)	PND2	PND21		
		Whole body	Serum	Liver	Brain
Lipidsa	0.86 (m); 0.90 (m); 1.26 (m); 1.30 (m)		–
Lactate	1.33 (d); 4.11 (q)	+	–	–
Glucose	3.54 (m); 3.66 (m); 3.70 (m); 3.74 (m); 3.78 (m); 3.82 (m); 3.86 (m); 3.90 (m)	+	+	–
Taurine	3.26 (t); 3.42 (t)		+	+
Cholinesb	3.20 (s); 3.22 (s); 3.23 (s)	+	+	–	–
Creatine	3.03 (s); 3.93 (s)	+
Glutamate	2.08 (m); 2.34 (m)			+	–
Glutamine	2.14 (m); 2.46 (m)				+
Glycine	3.55 (s)	+			+
Valine, leucine, isoleucine	0.94 (d); 0.96 (d); 1.0 (d); 1.01 (d); 1.05 (d)	–
Lysine	1.72 (m); 2.98 (m)	–
Glutathione	2.17 (m); 2.56 (m); 2.94 (m)			+
Glycogen	5.42 (m)			–
Aspartic acid	2.65 (dd); 2.80 (dd)				+
GABA	1.90 (q); 2.27 (t); 3.01 (t)				–
Abbreviations: +, significantly increased concentration; –, significantly decreased concentration; GABA, γ-aminobutyric acid. Chemical shifts (ppm) are relative to TMSP (1H, δ). Multiplicity of signals is indicated in parentheses: d, doublet; dd, doublet of doublet; m, multiplet; q, quadruplet; s, singlet; t, triplet. a Low-density lipoprotein and very-low-density lipoprotein. bPhosphatidylcholine and glycerophosphocholine.					

**Table 2:** Pairwise model parameter comparison of PND2 whole body and PND21 liver, brain, and serum between treatment groups.

Groups compared	n	PLS components	R2Y (%)	Q2 (cumulative)
PND2
Whole body
Control/0.025 µg BPA	38	2	99.3	0.979
Control/0.25 µg BPA	34	2	99.4	0.970
Control/25 µg BPA	31	1	99.5	0.989
0.025 µg BPA/0.25 µg BPA	31	3	98.0	0.731
0.025 µg BPA/25 µg BPA	29	1	98.3	0.943
0.25 µg BPA/25 µg BPA	25	2	98.9	0.969
PND21
Liver
Control/0.025 µg BPA	21	1	89.7	0.822
Control/0.25 µg BPA	24	1	99.5	0.980
Control/25 µg BPA	24	2	99.7	0.990
0.025 µg BPA/0.25 µg BPA	23	3	99.5	0.896
0.025 µg BPA/25 µg BPA	24	3	99.4	0.950
0.25 µg BPA/25 µg BPA	26	2	98.8	0.928
Brain
Control/0.025 µg BPA	22	2	99.0	0.955
Control/0.25 µg BPA	22	2	99.0	0.963
Control/25 µg BPA	21	1	90.0	0.664
0.025 µg BPA/0.25 µg BPA	23	3	98.5	0.932
0.025 µg BPA/25 µg BPA	25	3	99.5	0.941
0.25 µg BPA/25 µg BPA	26	4	99.4	0.895
Serum
Control/0.025 µg BPA	23	3	99.6	0.826
Control/0.25 µg BPA	25	4	98.9	0.932
Control/25 µg BPA	23	3	99.8	0.991
0.025 µg BPA/0.25 µg BPA	26	3	96.3	0.891
0.025 µg BPA/25 µg BPA	23	2	99.0	0.937
0.25 µg BPA/25 µg BPA	26	3	99.3	0.973

**Figure 2:**
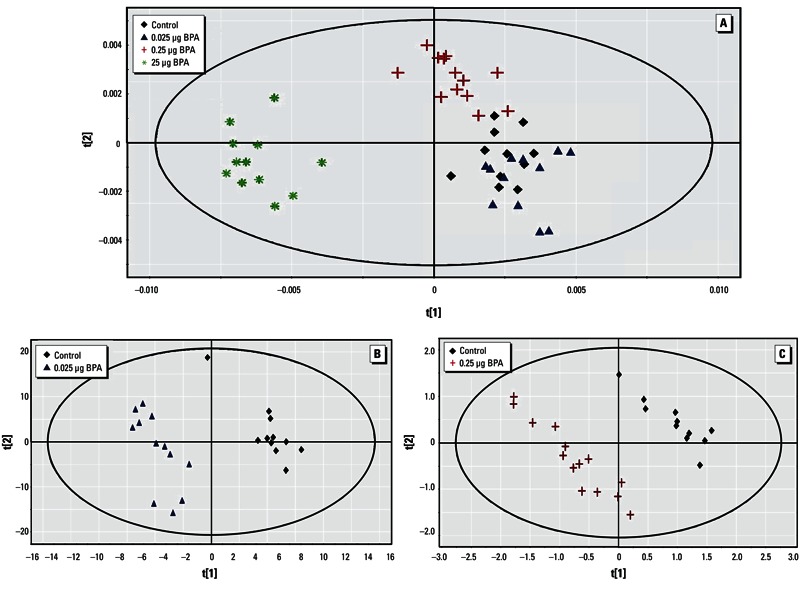
Two-dimensional PLS-DA score plot of integrated ^1^H NMR spectra of PND21 serum. (*A*) PLS‑DA results for all four treatment groups [control, *n* = 11; 0.025 µg BPA, *n* = 12; 0.25 µg BPA, *n* = 14; and 25 µg BPA, *n* = 12 (two components): R^2^Y = 55.3% and Q^2^ = 0.450]. (*B*) PLS‑DA results for control and 0.025 µg BPA (three components: R^2^Y = 99.6% and Q^2^ = 0.826) (*C*) PLS‑DA results for control and 0.25 µg BPA (four components: R^2^Y = 98.9% and Q^2^ = 0.932).

*PND21 serum.* The score plot of the PLS-DA using the entire data set (all groups taken together) showed a clear separation between the 25 µg BPA group and all other groups along the first latent component, and between the 0.25 µg BPA and 0.025 µg BPA groups (Figure 2A). This analysis generated a PLS-DA model with two latent components, with R^2^Y = 55.3% and Q^2^ = 0.450. Thirteen metabolites were identified as discriminant parameters in the metabolic profiles. Further pairwise comparisons between control and 0.025 µg BPA samples and between control and 0.25 µg BPA samples demonstrated a clear discrimination between the corresponding groups, and 14 and 11 metabolites, respectively, were identified as discriminant biomarkers (Figure 2B,C). BPA exposure (25 µg) induced a decrease in lipids [low-density lipoprotein (LDL) and very-low-density lipoprotein (VLDL)] and lactate concentrations and an increase in glucose, taurine, and cholines compared with controls (Table 1).

**Figure 3:**
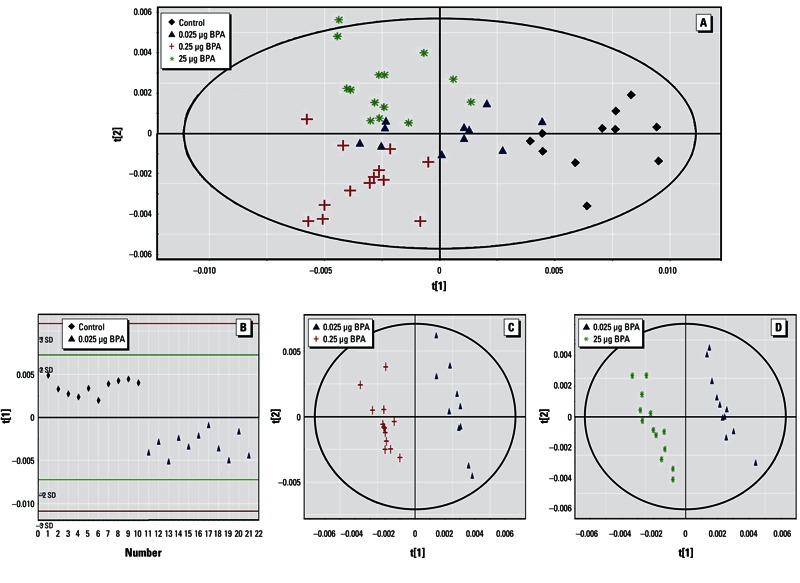
Two-dimensional PLS-DA score plot of integrated ^1^HNMR spectra of PND21 liver extracts. (*A*) PLS‑DA results for all four treatment groups [control, *n* = 11; 0.025 µg BPA, *n* = 11; 0.25 µg BPA, *n* = 13; 25 µg BPA, *n* = 14 (first and second latent variable of three components: R^2^Y 48.3% and Q^2^ = 0.421]. (*B*) PLS‑DA results for control and 0.025 µg BPA (one component: R^2^Y = 89.7% and Q^2^ = 0.822); red and green lines indicate ± 3 SD and ± 2 SD, respectively. (*C*) PLS‑DA results for 0.025 µg BPA and 0.25 µg BPA (three components: R^2^Y = 99.5% and Q^2^ = 0.896). (*D*) PLS‑DA results for 0.025 µg BPA and 25 µg BPA (three components: R^2^Y = 99.4% and Q^2^ = 0.950).

*PND21 liver.* The four-group comparison generated a PLS-DA model with two latent components, characterized by a correct representation of the data (R^2^Y = 48.3%) and Q^2^ equal to 0.421. The score plot of the PLS-DA showed a clear separation between control, 0.25 µg BPA, and 25 µg BPA, as well as between 0.25 µg BPA and 25 µg BPA (Figure 3A). The median of 46 buckets was significantly different according to the Kruskal–Wallis test, corresponding to three metabolites discriminating the three BPA-exposed groups, eight metabolites discriminating between 0.25 µg BPA and 25 µg BPA, and five metabolites separating control and 0.025 µg BPA from 0.25 µg BPA and 25 µg BPA (Table 2). For more specificity, pairwise comparisons were carried out between the 0.025 µg BPA group and all other groups, which led to the separations displayed in Figure 3B–D and further detailed in Table 1. When comparing 25 µg BPA samples with control samples, endogenous metabolite variations induced by BPA exposure showed increases in taurine, glutamate, and glutathione, as well as decreases in lactate, glucose, cholines, and glycogen (Table 1).

**Figure 4:**
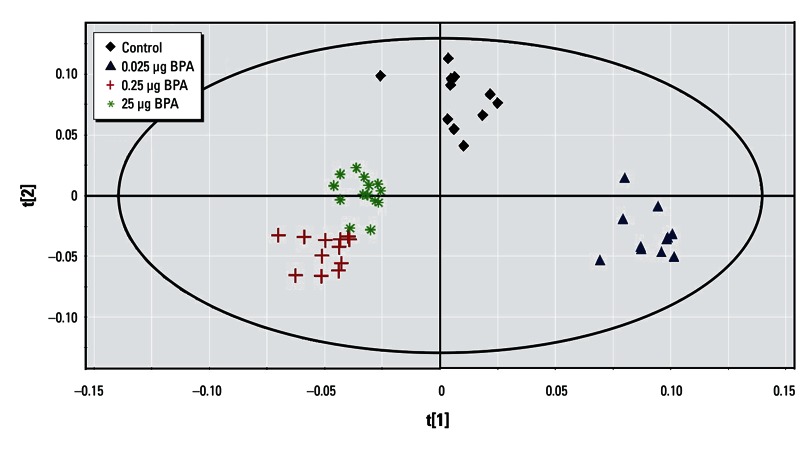
Two-dimensional PLS-DA score plot of integrated ^1^H NMR spectra of PND21 brain extracts from control (*n* = 11), 0.025 µg BPA (*n* = 11), 0.25 µg BPA (*n* = 13), and 25 µg BPA (*n* = 14) groups (first and second latent variable of three components: R^2^Y = 78.9% and Q^2^ = 0.564).

*PND21 brain.* The four-group comparison generated a PLS-DA model with three latent components, characterized by a faithful representation of the data (R^2^Y = 78.9%) and a good cumulative confidence criterion of prediction (Q^2^ = 0.564). The score plot of the PLS-DA showed a correct separation between controls and all BPA groups (Figure 4); 101 variables had a VIP value > 1.5, and the median of 76 buckets was statistically different according to the Kruskal–Wallis test, corresponding to 21 metabolites. Taken separately using pairwise comparisons, all groups could be discriminated from each other (Table 2). Endogenous metabolite variations between 25 µg BPA and control showed an increase in glutamine, glycine, and aspartic acid, and a decrease in cholines, glutamate, and γ-aminobutyric acid (GABA) (Table 1).

## Discussion

Developmental exposure to BPA affects the reproductive system and fertility, alters brain development and behavior, disrupts glucose homeostasis, and may contribute to the development of obesity and metabolic syndrome (Alonso-Magdalena et al. 2010; Cabaton et al. 2011; Richter et al. 2007; Ryan et al., 2010). In the present study, we examined whether a novel approach based on metabolomics profiling could detect subtle changes in the metabolome following BPA exposure at low to very low doses, and whether metabolic profiling could reveal differences that persist beyond the end of the exposure period. The BPA doses used in this study correspond to 1/2,000 to 1/2,000,000 of the no observed adverse effect level (NOAEL) for BPA (European Food Safety Authority 2006; U.S. Food and Drug Administration 2008), and the time of BPA exposure coincided with critical periods of development (GD8 to PND16).

Factors that affect fetal growth are also associated with the postnatal growth rate and with adult body weight in humans as well as in laboratory animals (Barker 2004; Coe et al. 2008). The classical tools of toxicology are not designed to detect the effects of an early exposure to low doses of endocrine disruptors. Recent developments in metabolomics allow us to further explore global changes in biological systems. NMR spectroscopy is broadly used in research to characterize metabolite structure. NMR spectroscopy fingerprinting, combined with multivariate statistical analysis, provides a powerful tool to detect metabolic changes induced by very low doses of EDs, allowing discrimination between several experimental groups on the basis of over- or underexpression of endogenous molecules. In the present study, we used ^1^H-NMR followed by PCA and PLS-DA analyses. PCA was used to detect potential outliers. PCA usually allows only a first-step discrimination between groups. When there are more than two groups, PLS-DA is more appropriate (Lindon et al. 2004). We performed PLS-DA—now routinely used in the field of metabolomics—to explore NMR fingerprints linked with perinatal exposure to BPA. Linear combinations of NMR buckets were constructed and then used to visualize differences (or similarities) between groups. One primary objective of our study was to identify exposure biomarkers that would correlate with the metabolic changes triggered by perinatal exposure to BPA, thus providing a proof of concept that ^1^H-NMR, completed by robust statistical analysis, is a powerful tool that would discriminate between exposed versus nonexposed animals.

PLS-DA is a classification method widely used in metabolomics to discriminate between treatment groups and to identify biomarkers responsible for this discrimination (Gavaghan et al. 2002; Martin et al. 2009). Our models were cross-validated, and their robustness was assessed with permutation tests. A robust model is associated with a Q^2^ value > 0.40 (McCombie et al. 2009). This was the case for all our models. The parameters of the PLS-DA models were estimated based on data sets comprising 49 (PDN21) to 63 (PND2) individuals. Because a large number of variables had to be processed, cross-validation was necessary to avoid overfitting. Results of permutation tests demonstrated that our analyses were robust and not obtained by chance. Discriminating variables were identified using the VIP and Kruskal–Wallis test, providing variables that explain the discrimination between groups. The next step was to connect some discriminant metabolites with possible mechanisms of action and to suggest some exposure biomarkers.

We were able to identify groups of pups that were perinatally exposed to low doses of BPA (0.025 µg/kg bw/day) with robust statistical models (Q_2_ > 0.4), even at a very early age (PND2). Among the discriminating variables, glucose was affected by perinatal exposure to BPA. Alonso-Magdalena et al. (2010) have also shown that BPA exposure to mice during pregnancy disrupts glucose homeostasis in 6-month-old male offspring. It makes sense that the shift observed for glucose would in turn be involved in the disruption of pyruvate biosynthesis through glycolysis. Pyruvate supplies energy to living cells through the Krebs cycle (aerobic respiration) and, alternatively, ferments to produce lactate (anaerobic respiration); pyruvate was also increased in PND2 pups. In this case, lactate could still be utilized by neurons, as already demonstrated in mice, rats, and humans (Wyss et al. 2011; Zilberter et al. 2010). At PND2, two other variables (increased levels of creatine and glycine) contributed to the discrimination between the control and 25 µg BPA groups, respectively. Creatine, a product of amino acid degradation (including glycine), is a major metabolite found mainly in muscle and brain, and it appears to be affected by prenatal exposure to BPA. An increase in creatine levels might lead to an increase in ATP production and, consequently, to increased energy metabolism. Glycine, a key precursor of porphyrins involved in heme production, is an inhibitory neurotransmitter in the central nervous system, especially in the spinal cord and brainstem, as well as in the retina. An increase in glycine may disrupt the global energy metabolism as well as brain and neurologic functions. Conversely, a decrease in essential amino acids, namely valine, leucine, and isoleucine, could reflect a disruption in their degradation pathways, which would be consistent with previous observations in GD18 rat fetuses exposed to butylbenzylphthalate (Sumner et al. 2009). Finally, we found that BPA exposure increased concentrations of cholines in PND2 mice, which may affect membrane integrity and could favor a decrease in acetylcholine production in the brain.

In PND21 tissues, we were able to distinguish all BPA exposure groups from controls. As observed in PND2 mice, glucose in serum and liver was affected by perinatal BPA exposure. Similarly, cholines were also decreased, reflecting a potential disruption in membrane integrity. In PND21 serum samples, a decrease in lipids (VLDL and HDL) and an increase in taurine (involved in the conjugation of bile acids) suggest that lipid metabolism may also be affected by BPA exposure. Taurine is a key amino acid for cardiovascular function and for the development and function of skeletal muscle. Taurine, also a major constituent of bile, was decreased in liver (and serum) in PND21 mice, reflecting a possible disruption in the digestive process. In liver extracts, an increase in glutathione in animals exposed to 25 µg BPA suggested a possible modulation of this detoxification pathway and also a hyperproduction of pyruvate, a key metabolite involved in the Krebs cycle, glycolysis, and glycogenesis. These changes may affect energy metabolism pathways.

Regarding PND21 brain extracts, recent studies have highlighted alterations in brain development following perinatal exposure to BPA (Itoh et al. 2012; Kunz et al. 2011). In the present study, two neurotransmitters (GABA and glutamate) were significantly decreased in all BPA-exposed animals compared with controls. Glutamate, the major excitatory transmitter in the brain, is the precursor for the synthesis of GABA, the major inhibitory transmitter in the adult brain. It is important to note that prior to their neurotransmission roles during adulthood, both glutamate and GABA are thought to influence processes of neural development, including proliferation, migration, differentiation, and survival (Lujan et al. 2005). Therefore, decreased levels of these neurotransmitters during postnatal development could be expected to exert lasting effects on the brain. In the present study, glutamine (the precursor of glutamate) was increased in the brain, whereas glutamate levels decreased, suggesting a conversion problem and possibly disruption and/or damage in brain function. Aspartate, produced from oxaloacetate (by transamination), is another neurotransmitter that stimulates *N*-methyl-d-aspartate receptors, the predominant molecule that controls synaptic plasticity and memory function. An increase in aspartate, known as an excitatory neurotransmitter, which increases the likelihood of depolarization in the postsynaptic membrane, might induce behavioral hyperactivity (Ishido et al. 2011).

## Conclusion

We observed significant and unequivocal shifts in the global metabolism of young male mice perinatally exposed to low doses of BPA. Our metabolomics studies on extracts from PND2 and PND21 males suggest that energy metabolism and brain function are the main targets. Work is ongoing on samples from female mice perinatally exposed to the same doses of BPA, euthanized at later time points with the aim of better understanding the mechanisms of action of BPA and the effects of BPA long after the time of exposure.

This study provides a proof of concept that metabolomics, here as NMR-based metabolic fingerprints, can be used as a novel approach and a powerful tool to detect metabolic shifts following perinatal exposure to BPA, even at very low doses. A similar approach could be used for other EDs, and we expect this approach to contribute to a better understanding of the modulations/disruption of the metabolome triggered during critical windows of development. Metabolomics could also be suitable and reliable for the analysis of biological fluids, such as urine or blood, in animal models and in human populations.

## Supplemental Material

(594 KB) PDF
