# Supplementary material for: Effects of Low Doses of Bisphenol A on the Metabolome of Perinatally Exposed CD-1 Mice
Source: Environ Health Perspect. 2013 Feb 21;121(5):586–93. doi: 10.1289/ehp.1205588 (PMC3673190; doi:10.1289/ehp.1205588)
Supplement: (594 KB) PDF [file ehp.1205588.s001.pdf]

Supplemental Material

Low Doses of Bisphenol-A Disrupt the Metabolome in Perinatally Exposed CD-1 Mice

Nicolas J. Cabaton[1,2], Cécile Canlet[1,2], Perinaaz R. Wadia[3], Marie Tremblay-Franco[1,2], Roselyne Gautier[1,2], Jérôme Molina[1,2], Carlos Sonnenschein[3], Jean-Pierre Cravedi[1,2], Beverly S. Rubin[3], Ana M. Soto[3], and Daniel Zalko[1,2]

1- INRA, UMR 1331 TOXALIM (Research Centre in Food Toxicology), 31027 Toulouse, France.

2- Toulouse University, INP, UMR 1331 TOXALIM, 31000 Toulouse, France.

3- Department of Anatomy and Cellular Biology, Tufts University School of Medicine, Boston, Massachusetts 02111 USA.

Corresponding author:

Dr Daniel Zalko

INRA, UMR 1331 TOXALIM (Research Centre in Food Toxicology), 180 Chemin de Tournefeuille, BP 93173, 31027 Toulouse Cedex 3, France.

Tel: +33 561 285 004, Fax: +33 561 285 244, E-mail: dzalko@toulouse.inra.fr

Table of contents

Supplemental material, Figure S1: PCA score plot of PND2……………………………...……......2

Supplemental material, Figure S2: PCA score plot of PND21 Serum..……………...……......3

Supplemental material, Figure S3: PCA score plot of PND21 Liver……………….…..……..4

Supplemental material, Figure S4: PCA score plot of PND21 Brain ..…………………..…....5

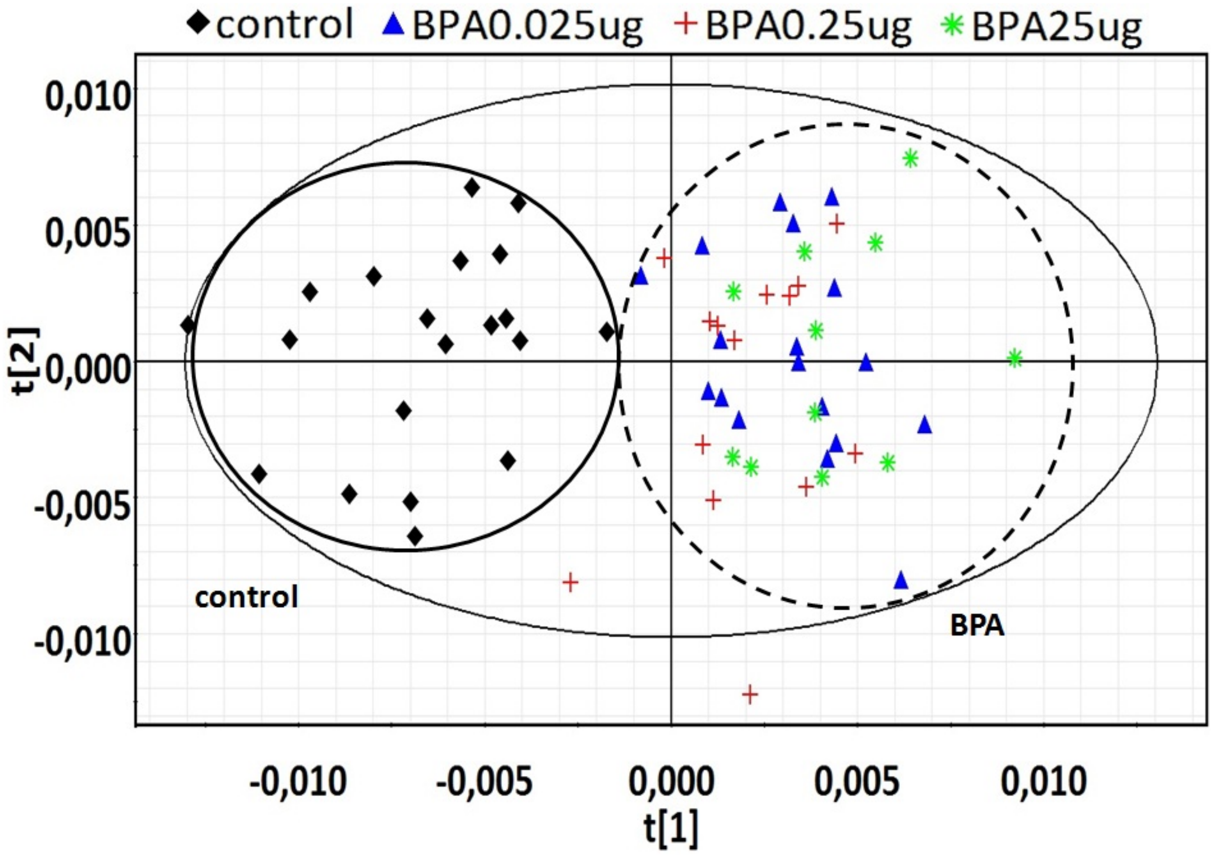

Supplemental Material, Figure S1: Two-dimensional PCA scores plot of PND2 whole body F1 male extracts integrated ^{1}H-NMR spectra for Control (N=20), BPA0.025µg/kg (N=18), BPA0.25µg/kg (N=14), BPA25µg/kg (N=11) (1st and 2nd principal components out of 15 components: R^2X=38.4% out of 93.3%).

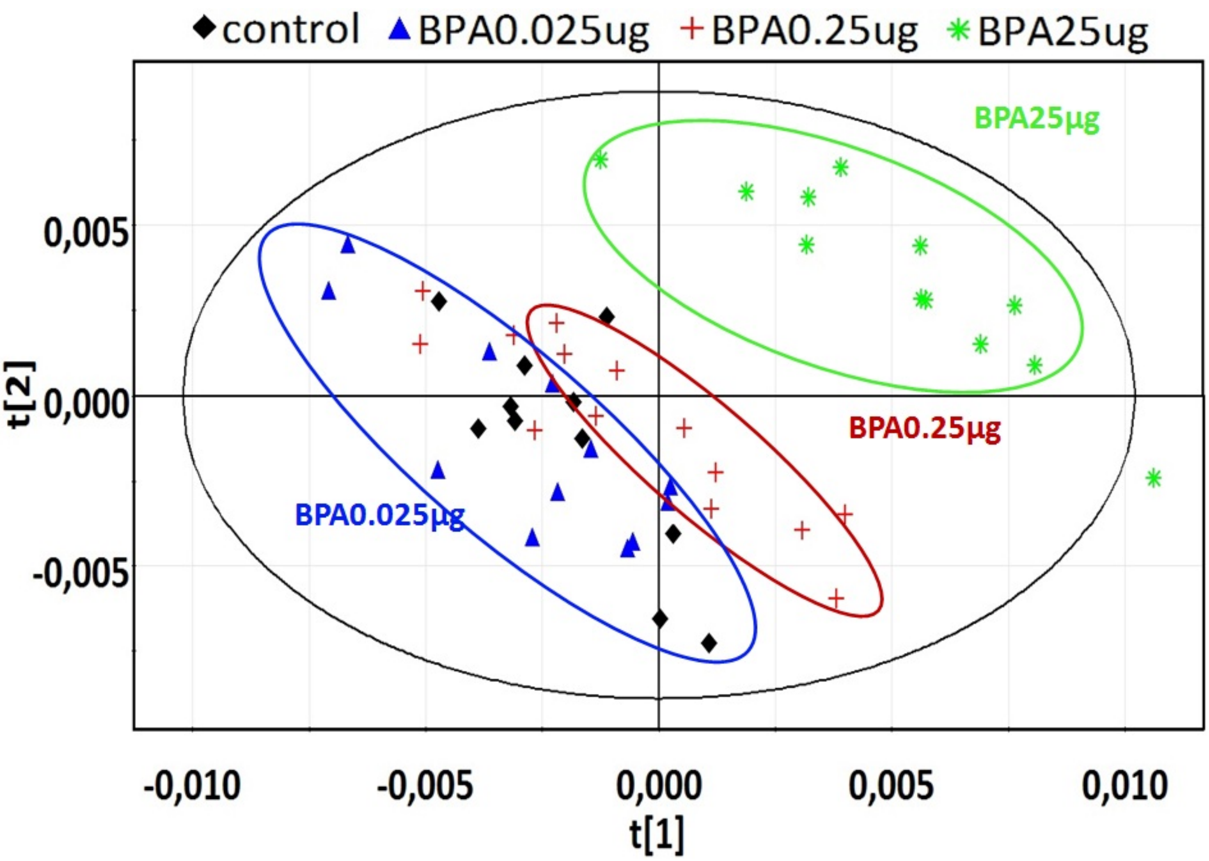

Supplemental Material, Figure S2: Two-dimensional PCA scores plot of PND21 serum F1 male integrated ^{1}H-NMR spectra for Control (N=11), BPA0.025µg (N=12), BPA0.25µg (N=14) and BPA25µg (N=12) (1st and 2nd principal components out of 11 components: R^2X=40.4% out of 50.3%).

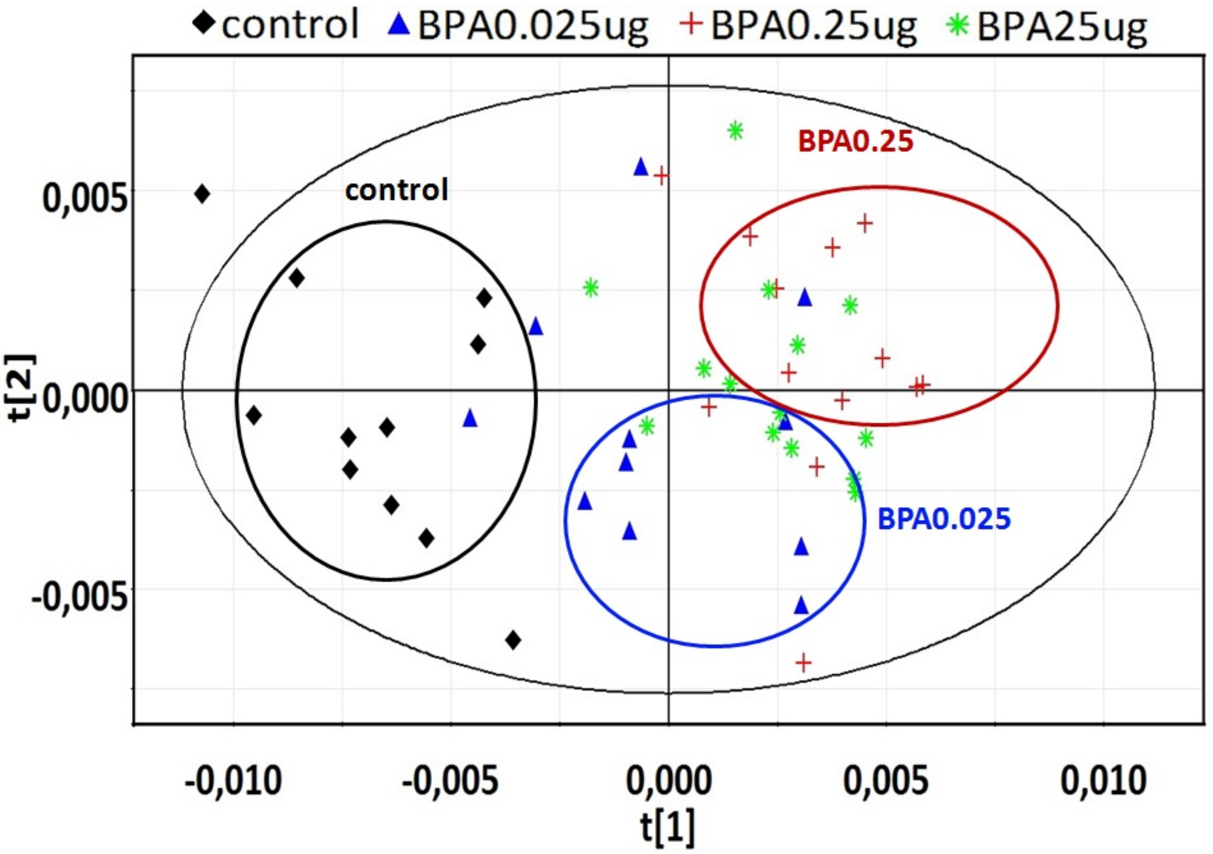

Supplemental Material, Figure S3: Two-dimensional PCA scores plot of PND21 liver F1 male extracts integrated ^{1}H-NMR spectra for Control (N=11), BPA0.025µg (N=11), BPA0.25µg (N=13), BPA25µg/kg (N=14) (1st and 2nd principal components out of 8 components: R^2X=45.3% out of 88.6%).

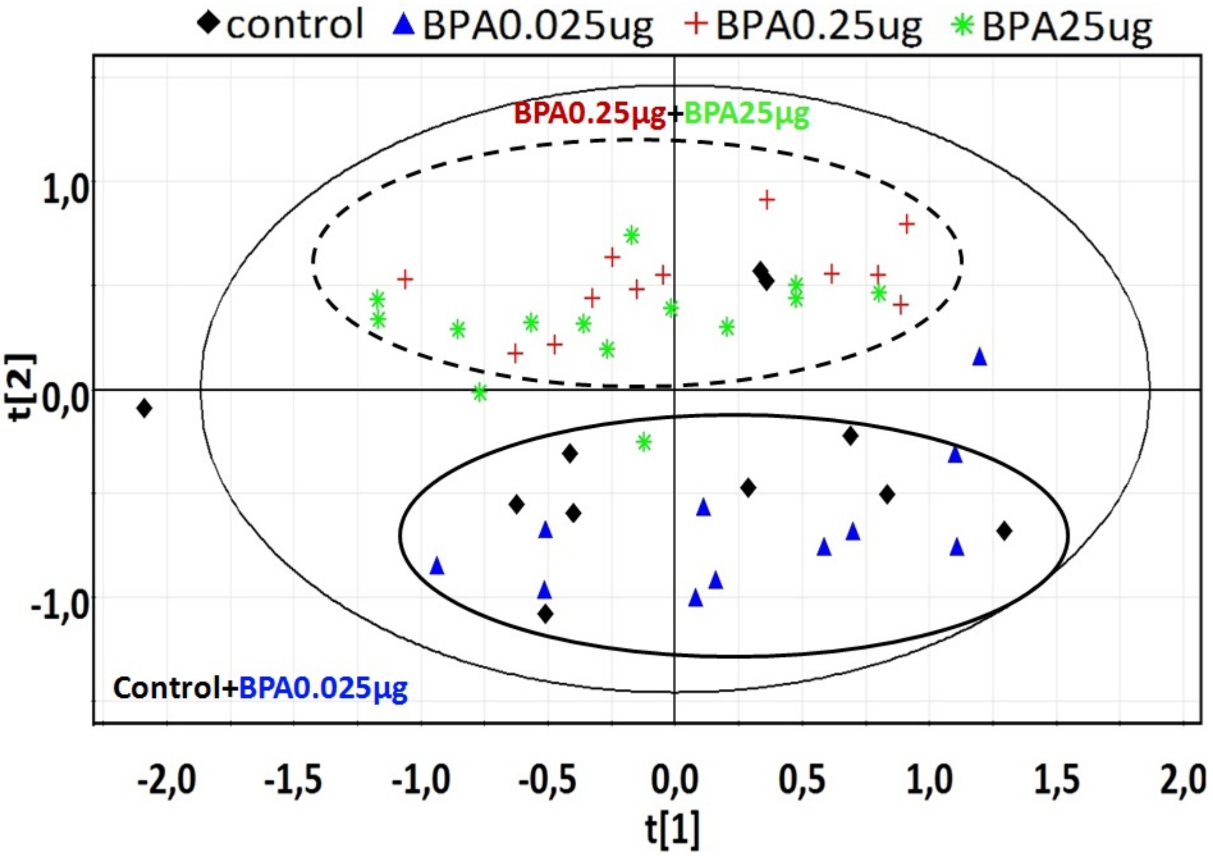

Supplemental Material, Figure S4: Two-dimensional PCA scores plot of PND21 brain F1 male extracts integrated ^{1}H-NMR spectra for Control (N=11), BPA0.025µg (N=11), BPA0.25µg (N=13), BPA25µg/kg (N=14) (1st and 2nd principal components out of 11 components: R^2X=25.9% out of 74.6%).
